# Retraction Note: Disruption of the Bcr-Abl/Hsp90 protein complex: a possible mechanism to inhibit Bcr-Abl-positive human leukemic blasts by novobiocin

**DOI:** 10.1038/s41375-020-0823-z

**Published:** 2020-04-08

**Authors:** L. X. Wu, J. H. Xu, K. Z. Zhang, Q. Lin, X. W. Huang, C. X. Wen, Y. Z. Chen

**Affiliations:** 1 Deparment of Pharmacology, Institute of Clinical Pharmacology, Fujian Medical University, Fuzhou, PR China; 2 Fujian Institute of Hematology, Union Hospital, Fujian Medical University, Fuzhou, PR China

Correction to: *Leukemia*

10.1038/leu.2008.89

The authors have retracted this article [1]. An investigation by Fujian Medical University identified multiple errors in the assembly of the figures, most notably in the duplication of loading controls. All authors agree with this retraction.
